# Cancer‐associated fibroblasts‐secreted lactate promotes RNA polymerase III subunit G‐mediated epithelial–mesenchymal transition in non‐small cell lung cancer by increasing m6A modification of zinc finger protein 384

**DOI:** 10.1002/ccs3.70037

**Published:** 2025-07-25

**Authors:** Ping Li, Xing Yang, Hao Tang, Zhiping Zhou, Bin Liu

**Affiliations:** ^1^ Department of Respiratory and Critical Care Medicine Hunan Aerospace Hospital Changsha Hunan China

**Keywords:** cancer‐associated fibroblasts, epithelial‐mesenchymal transition, non‐small cell lung cancer, POLR3G, ZNF384

## Abstract

Most advanced non‐small cell lung cancer (NSCLC) patients have metastasis, which poses great risks to their survival. As the most abundant components in the tumor microenvironment (TME), cancer‐associated fibroblasts (CAFs) can induce epithelial–mesenchymal transition (EMT) to promote tumor. This study aimed to explore the potential molecular mechanisms of CAFs‐mediated EMT in NSCLC. The gene expression was assessed using RT‐qPCR, immunofluorescence, and Western Blot. Cells phenotypes were evaluated through CCK‐8, scratch, and transwell assays, respectively. Lactate levels were measured with a commercial kit. The m6A level of zinc finger protein 384 (ZNF384) was measured using methylated RNA immunoprecipitation. The molecular interactions was checked using chromatin immunoprecipitation and dual luciferase reporter assay. ZNF384 was upregulated in NSCLC. ZNF384 knockdown suppressed NSCLC cell proliferation and inhibited EMT‐related protein vimentin and Snail, but elevated E‐Cadherin. Mechanistically, CAFs‐secreted lactate promoted the H3K18 lactylation of methyltransferase‐like 3 (METTL3) promoter region and further increased the m6A modification of ZNF384. ZNF384 promoted the transcription of RNA polymerase III subunit G (POLR3G) by binding to POLR3G promoter region. CAFs induced EMT in NSCLC cells by enhancing ZNF384 expression. Additionally, POLRG3 silencing counteracted the promoting effect of ZNF384 overexpression on EMT in NSCLC. CAFs facilitating cell proliferation and EMT by modulating the METTL3/ZNF384/POLR3G axis. It is suggested that CAFS‐related TME could be an approach for treating NSCLC.

## Graphical abstract

In the tumor microenvironment of non‐small cell lung cancer, lactate secreted by cancer‐associated fibroblasts enhances the lactylation of H3K18 within the promoter region of methyltransferase‐like 3 (METTL3). Furthermore, METTL3 promotes the m6A modification of zinc finger protein 384 (ZNF384) to increase its stability. The upregulation of ZNF384 transcriptionally activates polymerase III subunit G, which in turn promotes the epithelial–mesenchymal transition in cancer cells, thereby facilitating tumor progression.

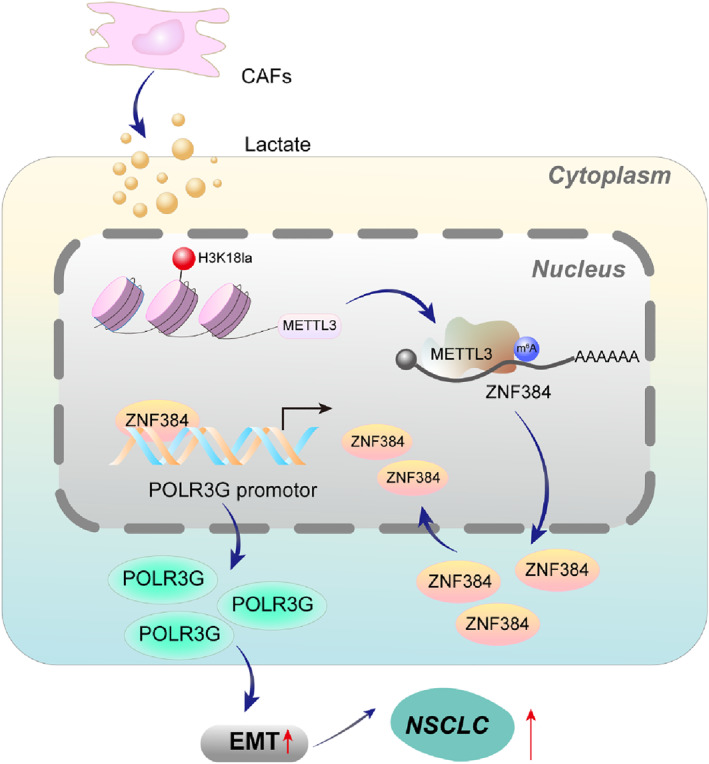

## 1 INTRODUCTION

Lung cancer poses a major health issue worldwide, marked by high morbidity and mortality rates.^1^ Non‐small cell lung cancer (NSCLC) accounts for over 80% of all lung cancer cases.^1^ Since early‐stage NSCLC typically exhibits no apparent symptoms, most patients are diagnosed at an advanced stage and are difficult to treat surgically.^2^ In cases of advanced NSCLC, metastasis emerges as a critical factor contributing to poor prognosis and even causing death.^3^ Current clinical approaches, including radiotherapy and chemotherapy, are often associated with issues such as adverse side effects and drug resistance.^4^ Consequently, it is crucial to investigate the molecular processes involved in the distant metastasis of NSCLC to enhance patient survival rates.

As an essential tumor microenvironment (TME) component, cancer‐associated fibroblasts (CAFs) interact with tumor cells and release various growth factors and cytokines that greatly impact tumorigenesis,^5^ such as colorectal cancer,^6^ gastric cancer,^7^ and pancreatic cancer.^8^ Recent studies have identified CAFs as an independent prognostic indicator for NSCLC patients, in which CAFs are significantly correlated with unfavorable outcomes and reduced survival rates.^9^ It has been reported that the transcription factor PRRX1 supports CAFs‐mediated lung cancer development by upregulating OLR1 expression.^10^ Meng et al. identified a subset of senescence‐like CAFs that enhance NSCLC cell growth and confer resistance to radiotherapy via the Janus kinase/signal transducer and activator of transcription (JAK/STAT) pathway.^11^ More importantly, CAFs also participated in the epithelial–mesenchymal transition (EMT) of tumor cells.^12^ EMT enables epithelial cells to adopt mesenchymal traits, thereby increasing their migratory and invasive potential to facilitate tumor metastasis.^13^ Wang et al. revealed that SDF‐1 produced by CAFs promoted the EMT of lung adenocarcinoma cells by upregulating CXCR4, β‐catenin and PPARδ.^14^ CAFs interacted with NSCLC cells through paracrine signals to enhance their EMT process and contribute to tumor metastasis.^15^ However, the specific molecular mechanism by which CAFs regulate the EMT process in NSCLC remains inadequately understood, necessitating further research.

Methyltransferase‐like 3 (METTL3) is a methyltransferase that specifically mediates N6‐Methyladenosine (m6A) modification.^16^ Ma et al. reviewed the critical role and potential mechanisms of METTL3 in the progression of lung cancers.^17^ For instance, METTL3 promoted the progression of NSCLC by increasing the m6A levels and subsequent translation of yes‐associated protein.^18^ Do METTL3 and CAFs have a regulatory relationship, then? Research indicated that lactate accumulation within the TME can lead to the upregulation of METTL3 through H3K18 lactylation.^19^ Concurrently, it has been reported that CAFs secrete lactate into the TME via the glycolytic pathway.^20^ Therefore, we hypothesize that CAFs may upregulate METTL3 in NSCLC cells by elevating its lactylation at H3K18.

Zinc finger protein 384 (ZNF384) can bind consensus DNA sequences and functions as a transcription factor in tumorigenesis.^21^ Specifically, ZNF384 was upregulated and transcriptionally activated ZEB1 to facilitate EMT in breast cancer.^21^ Additionally, ZNF384 was highly expressed and enhanced the invasive and migratory capabilities of colorectal cancer cells by regulating MMP2.^22^ Analysis of the Starbase database indicated that ZNF384 was elevated in lung cancer, but its exact role in NSCLC is still being elucidated. Surprisingly, research studies have indicated that METTL3 mediated m6A modification of ZNF384, which in turn stabilized ZNF384 mRNA and facilitated the advancement of hepatocellular carcinoma.^23^ However, further investigation is needed to determine whether m6A‐modified ZNF384 can regulate EMT in NSCLC.

RNA polymerase III subunit G (POLR3G) is a component of RNA polymerase III (Pol III), and its overexpression in various oncological contexts means adverse outcomes for patients.^24^ Low expression of POLR3G is correlated with high survival rates in lung adenocarcinoma patients.^25^ In addition, POLR3G promotes cell migration and EMT in bladder cancer.^26^ Nevertheless, the regulatory role of POLR3G in the EMT process of NSCLC remains unexplored and warrants further investigation.

Additionally, JASPAR database (https://jaspar.elixir.no/) has revealed potential binding sites between ZNF384 and POLR3G promoter, suggesting that ZNF384 may interact with POLR3G to regulate its transcriptional activity. Drawing from the aforementioned research and bioinformatics data analysis, we supposed that lactate released by CAFs increased METTL3 expression via H3K18 lactylation, and upregulation of METTL3 promoted the m6A modification of ZNF384 to activate POLR3G transcription, thereby facilitating cells migration and EMT in NSCLC. These findings will provide novel insights into the metastatic mechanisms underlying NSCLC and contribute to developing therapeutic strategies.

## 2 MATERIALS AND METHODS

### 2.1 Human specimens

The tumor and adjacent paracancerous tissue samples from 20 NSCLC patients were obtained during the surgery under a protocol approved by the Hunan Aerospace Hospital (No. HNHTYY20240918LLSH‐015‐01). All participants were informed of the purpose and risks of this study and provided informed consent. The basic information and clinicopathological parameters of all patients were complete, and no radiotherapy or chemotherapy was performed before surgery. The collected samples were used for the separation of CAFs, and the remaining tissues were stored at −80°C for subsequent experiments.

### 2.2 Isolation of fibroblast

This protocol for isolating CAFs is based on the paper by Zawieracz and Eckert.^27^ The minced tumor tissue was placed into a conical tube containing sterile phosphate buffered saline (PBS), 0.25% trypsin (15090046, Gibco), and 0.1% EDTA (AM9260G, Gibco). The tube was positioned on an orbital shaker at 37°C for 30 min. Following the removal of the supernatant, the tissue was incubated with a dissociation solution containing PBS, hyaluronidase (9001‐54‐1, Sigma‐Aldrich), and type 3 collagenase (9007‐34‐5, Sigma‐Aldrich) at 37°C for 6 h. Subsequently, the supernatant containing the dissociated cells was carefully centrifuged for 5 min. The resulting pellet was washed with Roswell Park Memorial Institute 1640 (RPMI 1640) medium containing fetal bovine serum‌ (FBS) and penicillin‐streptomycin solution. The fibroblasts were resuspended in the above culture medium and inoculated into a dish. The procedure for isolating normal fibroblasts (NF) from adjacent paracancerous tissues closely resembles the previously described methodologies, and the culture conditions employed are the same as those utilized for CAFs. Passage culture was performed when CAFs and NF were observed to reach 80%–100% confluence.

### 2.3 Cell culture and treatment

The normal human lung epithelial cell line BEAS‐2B and lung cancer cell lines, including A549, H1299, HCC827, and H23, were obtained from the American Type Culture Collection (ATCC). H1299, HCC827, and H23 cells were cultured in RPMI 1640 medium (Gibco) containing 10% FBS (A5670701, Gibco) and 1% penicillin–streptomycin solution (15070063, Gibco), whereas A549 cells were cultured in Ham's F12K medium (21127030, Gibco) and dulbecco's modified eagle medium (11966025, Gibco) was used to maintain BEAS‐2B cell growth. All cell lines were placed in a humidified incubator at 37°C with a 5% CO_2_ in air atmosphere.

To investigate the impact of CAFs on the malignant behaviors of NSCLC cells, the cultured medium of CAFs and NFs was collected separately, and they were treated with NSCLC cells as a conditional medium. A549 and H1299 cells were pretreated with the lactate production inhibitor oxamate (10 μM, 565‐73‐1, MedChemExpress) for 24 h and then replaced with fresh culture medium.

### 2.4 Cell transfection

The overexpression plasmid for ZNF384 and the short hairpin RNA against METTL3, ZNF384, and POLR3G expression were synthesized by GenePharma. All plasmids, as well as their corresponding controls, were packaged with lentivirus and subsequently transfected into A549 and H1299 cells with Lipofectamine 3000 (L3000150, Invitrogen) according to the protocols. Following a 24‐h transfection period, puromycin was employed to select the successfully transfected cells, and then cells were harvested to assess transfection efficiency for subsequent experiments.

### 2.5 RNA extraction and quantitative reverse transcription‐polymerase chain reaction

To extract total RNA from tissues and cells, Trizol reagent (R411‐02, Vazyme), chloroform (75915E, Adamas) and isopropyl alcohol (75885AU, Adamas) were applied sequentially. The HiScript cDNA Synthesis Kit (R212‐01, Vazyme) was used to reverse‐transcribed the collected RNA sample into cDNA. According to the instructions of the SuperReal PreMix Plus kit (FP205‐01, TIANGEN), a 20 μL reaction system was prepared, and 40 cycles of two‐step PCR reactions (95°C for 10s and 60°C for 30s) were carried out after the pre‐denaturation period. The relative quantitative value of each gene was assessed using the 2^−ΔΔCT^ formula, with β‐actin acting as the internal control gene. The primer sequences used in this study are as follows:

ZNF384 forward:GGTAGAATGGAAGAATCTCACTTCA,

ZNF384 reverse:CTGTCAGCAAGGTGGGGTAG;

METTL3 forward:GAGTGCATGAAAGCCAGTGA,

METTL3 reverse:CTGGAATCACCTCCGACACT;

POLR3G forward:CCGTTAAGAGCCTTCTTT,

POLR3G reverse:CTCATGCTGGTAAAGCAACC;

β‐actin forward:CTCTTCCAGCCTTCCTTCCT,

β‐actin reverse:AGCACTGTGTTGGCGTACAG.

### 2.6 Western Blot

Total protein from tissues and cells was extracted using radio immunoprecipitation assay buffer and quantified by the bicinchoninic acid protein concentration assay kit (P0010S, Beyotime). The protein sample was mixed with an appropriate loading buffer and placed in a boiling water bath for 10 min. Then, the denatured protein sample was added evenly into the prepared sodium dodecyl sulfate polyacrylamide gel electrophoresis gel for electrophoresis. After separating the protein, the substances on the gel were transferred to a polyvinylidene fluoride membrane, and the membrane was blocked with 5% skim milk. Subsequently, primary antibodies of ZNF384 (ab176689, Abcam), METTL3 (ab195352, Abcam), E‐Cadherin (ab314063, Abcam), vimentin (ab92547, Abcam), Snail (ab216347, Abcam), POLR3G (ab200721, Abcam), Kla (PTM‐1401RM, PTMBio), and H3K18la (PTM‐1406RM, PTMBio) were incubated with the membranes overnight at 4°C. Then, the protein bands were treated with HRP‐labeled secondary antibody for 1 hour incubation. An electrochemiluminescence luminescent reagent was applied for visualization under a multi‐functional imaging system, and Image‐J software was used for analysis.

### 2.7 CCK‐8 assay

A549 and H1299 cells were inoculated into a 96‐well plate. Following this pre‐culture period, the cells were subjected to specific treatments and maintained in culture for 24 h. Then, CCK‐8 solution (C0037, Beyotime) was introduced to the designated wells, and the plates were incubated for an additional hour. Subsequently, a microplate reader (Thermo Scientific) was employed to measure the absorbance of the wells at 450 nm, facilitating the calculation of cell viability.

### 2.8 Scratch test

First, use a marker to draw some lines on the back of the six‐well plate to mark it. Then, the cells were seeded into the wells. The following day, a trace of the center of the cell was spread using a sterile pipette tip, and then rinsed with PBS. After an incubation of 24 h, the cell migration rate was determined by observing the migration of cells under an optical microscope.

### 2.9 Transwell assay

The Matrix‐Gel™ (C0371, Beyotime) was uniformly applied to the base of the Transwell chamber (Corning). The appropriately treated cells were inoculated into the Transwell chamber, followed by the addition of a serum‐free medium. Then, the lower side of the chamber was fulfilled with 500 μL complete culture medium. The chamber was then carefully positioned into the 24‐well plate and incubated at 37°C for 24 h. After the incubation, 600 μL of 4% paraformaldehyde fixative (P0099, Beyotime) was added to the clean wells of the 24‐well plate, and the chamber was immersed in the fixation solution for 30 min. After washing with PBS, 600 μL of 1% crystal violet staining solution was applied for a staining period of 10 min. Once adequately air‐dried, cell invasion was assessed using an optical microscope (Leica).

### 2.10 Immunofluorescence

The CAFs were seeded into a 24‐well plate, and after washing with PBS, a 4% paraformaldehyde solution was applied for a fixation period of 15 min. The fixative was discarded, and CAFs were treated with Triton‐X‐100 solution (P0096, Beyotime) for 20 min. Subsequently, CAFs were blocked with a 5% bovine serum albumin (37520, Thermo Scientific) for 60 min. After washing with PBS, CAFs were mixed with anti‐α‐SMA (A2547, Sigma‐Aldrich) and anti‐FSP1 (ab219986, Abcam) overnight at 4°C. Following a rinse with PBS, fluorescently labeled secondary antibodies were introduced for a 60 min incubation in the dark. Then, CAFs were stained with 4’,6‐diamidino‐2‐phenylindole for 5 min, and PBS was applied to eliminate excess dye. Finally, a fluorescence microscope (Thermo Scientific) was applied to observe the samples.

### 2.11 Detection of lactic acid level

The lactic acid kit utilized in this study was purchased from Enzyme‐linked (ml076587) and was evaluated according to the instructions. Briefly, the harvested cells were subjected to sonication in an extraction solution, followed by centrifugation to isolate the supernatant. Subsequently, the appropriate reagents were added to both the test sample and the standard, and the mixture was incubated at 37°C for 30 min. The OD530 was measured by a spectrophotometer (METTLER TOLEDO), and the lactic acid concentration was calculated based on the established formula.

### 2.12 Methylated RNA immunoprecipitation

Total RNA was extracted using the TRIzol reagent (15596018CN, Invitrogen). Then, the quality and quantity of total RNA were analyzed, and the RNA was cleaved into oligonucleotides of approximately 100 nucleotides after purification. Then, a m6A‐specific antibody (No. 202003, Synaptic Systems) was mixed with cleaved RNA and protein A/G beads to co‐incubate. The beads were then eluted with free m6A (Sigma‐Aldrich), and the eluted solution obtained was analyzed using qPCR experiments to determine enrichment.

### 2.13 Chromatin immunoprecipitation (ChIP)

Pierce Magnetic Chromatin Immunoprecipitation (ChIP) Kit (Thermo Scientific) was used to perform the ChIP assay under the manufacturer's instructions. Briefly, cells were crosslinked using 1% formaldehyde; then, a halt cocktail was added to the mixture. The next step is to treat the collected cell pellet with an MNase digestion buffer solution to obtain the supernatant containing digested chromatin. Subsequently, the supernatant was incubated with the antibody against ZNF384 (ab176689, Abcam) or H3K18la (PTM‐1406RM, PTMBio) and Protein A/G Magnetic Beads to complete immunoprecipitation. The eluted IP solution was obtained through repeated elution of the beads, DNA recovery was performed to acquire purified DNA, and the final analysis was conducted by qPCR.

### 2.14 Dual‐luciferase reporter assay

The mutant fragment POLR3G containing the two previously predicted binding sites (Pro#1 and Pro#2) was cloned into the pGL3 basic vector (Promega) to obtain different dual‐luciferase reporter plasmids. Subsequently, the above constructed plasmid was co‐transfected with OE‐ZNF384 or OE‐NC plasmid into A549 or H1299 cells. The Dual‐Glo system (Promega) was applied to quantify the relative luciferase activity 48 h after transfection and the luciferase signal in the assay results were compared and examined with the endogenous luciferase signal.

### 2.15 Statistical analysis

The data statistics and analysis in this study utilized GraphPad Prism 9.0 software and were presented as mean ± standard deviation. Specifically, an independent samples *t*‐test was employed for comparisons between two groups, while one‐way analysis of variance followed by Tukey's test was used for comparisons among multiple groups. All experiments were conducted in triplicate or more, and a *p*‐value of less than 0.05 was deemed statistically significant.

## 3 RESULTS

### 3.1 ZNF384 promoted EMT in NSCLC cells

**FIGURE 1:**
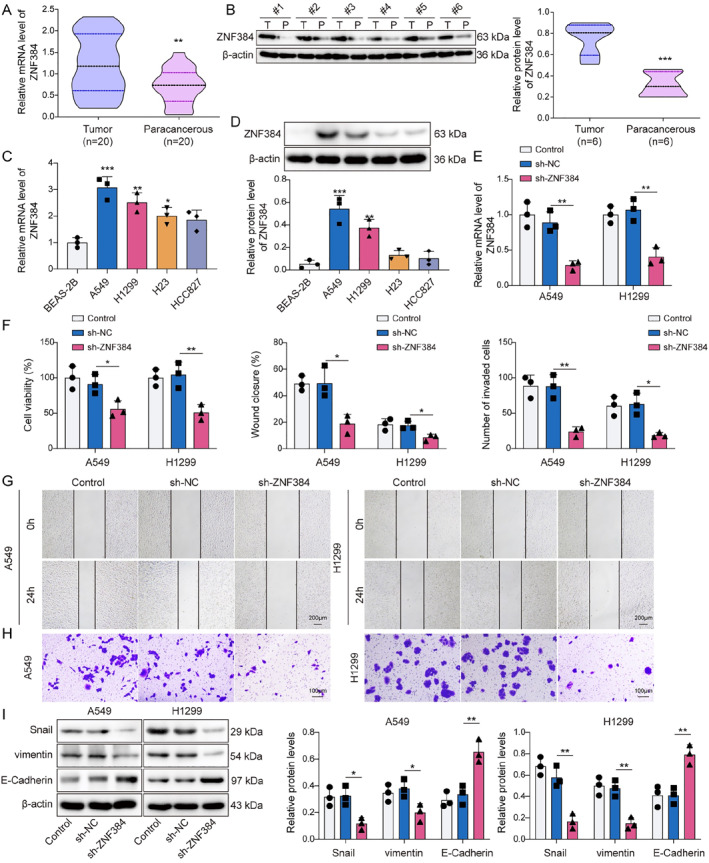
ZNF384 promoted epithelial–mesenchymal transition in non‐small cell lung cancer cells. (A and B) RT‐qPCR and Western blot were performed to determine ZNF384 expression in clinical tissues. (C and D) RT‐qPCR and Western blot were conducted to detect ZNF384 expression in BEAS‐2B, A549, H1299, HCC827, and H23 cell lines. sh‐NC or sh‐ZNF384 was transfected into A549 and H1299 cells for 24 h. (E) RT‐qPCR was applied to quantify ZNF384 expression. (F) CCK‐8 assay evaluated the viability of A549 and H1299 cells. (G and H) Scratch test and transwell assay measured A549 and H1299 cell migration and invasion. (I) Western blot was used to assess E‐Cadherin, vimentin, and Snail expression. *p < 0.05, **p < 0.01, ***p < 0.001.

First, we discovered that ZNF384 levels were markedly higher in NSCLC tissues compared to those in paracancerous tissues (Figure 1A,B). Additionally, ZNF384 presented the higher expression in A549 and H1299 cells among all lung cancer cell lines (Figure 1C,D). Therefore, we chose A549 and H1299 cell lines for the subsequent experiments and performed transfection with sh‐ZNF384 to knock down ZNF384. As illustrated in Figure 1E, the transfection of sh‐ZNF384 markedly inhibited ZNF384 levels. Then, we examined the influence of ZNF384 knockdown on NSCLC cell function. As displayed in Figure 1F–H, the silencing of ZNF384 resulted in a reduction of cell viability in migration and invasion. In addition, the levels of EMT marker proteins, specifically vimentin and Snail, exhibited a reduction corresponding to the diminished expression of ZNF384 (Figure 1I). Conversely, the knockdown of ZNF384 resulted in an upregulation of E‐Cadherin (Figure 1I). These results indicated that ZNF384 could facilitate EMT in NSCLC cells.

### 3.2 CAFs increased ZNF384 m6A modification by enhancing METTL3 H3K18 lactylation

**FIGURE 2:**
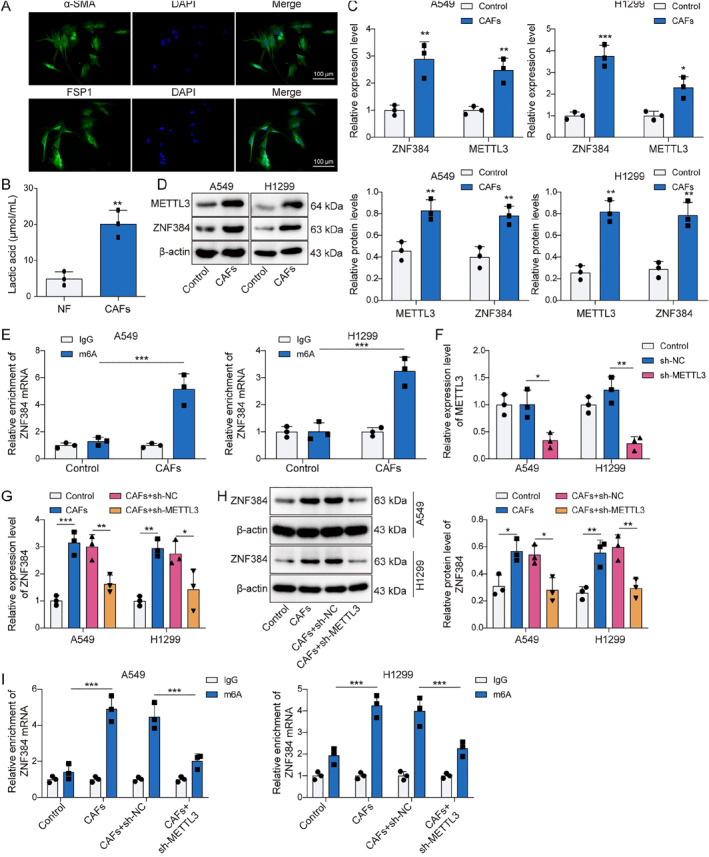
Cancer‐associated fibroblasts (CAFs) increased ZNF384 m6A modification by enhancing methyltransferase‐like 3 (METTL3) H3K18 lactylation. CAFs were isolated from non‐small cell lung cancer tissues. (A) Representative immunofluorescence images of α‐SMA and FSP1 in CAFs. (B) Lactate production was measured by a lactic acid test kit. A549 and H1299 cells treated with conditioned medium (CM) from CAFs and NFs. (C and D) RT‐qPCR and Western blot were performed to determine ZNF384 and METTL3 expression. (E) Methylated RNA immunoprecipitation (MeRIP) assay was applied to detect the m6A levels for ZNF384. sh‐NC or sh‐METTL3 was transfected into A549 and H1299 cells for 24 h (F) METTL3 expression was determined by RT‐qPCR. A549 and H1299 cells were treated with CM from CAFs or NFs and were transfected with sh‐METTL3 or sh‐NC. (G and H) RT‐qPCR and Western blot were conducted to evaluate ZNF384 expression. (I) MeRIP assay was applied to detect m6A modification levels of ZNF384. A549 and H1299 cells were co‐cultured with CAFs or NFs for 24 h. *p < 0.05, **p < 0.01, ***p < 0.001.

**FIGURE 3:**
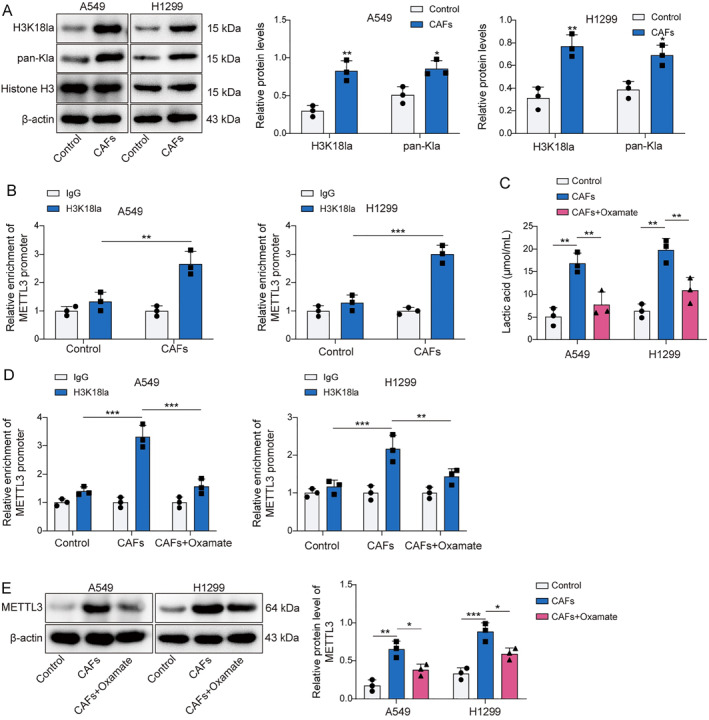
Cancer‐associated fibroblasts (CAFs) increased ZNF384 m6A modification by enhancing methyltransferase‐like 3 (METTL3) H3K18 lactylation. (A) Western blot determined the levels of Pan Kla and H3K18la. (B) The H3K18la level on METTL3 promoter was detected by ChIP assay. A549 and H1299 cells were pretreated with Oxamate and co‐cultured with CAFs or NFs. (C) The lactate levels in the supernatant of normal fibroblasts, CAFs and CAFs treated with Oxamate were detected by a lactic acid test kit. (D) The H3K18la level on METTL3 promoter was detected by ChIP assay. (E) Western blot was applied to evaluate METTL3 protein level. *p < 0.05, **p < 0.01, ***p < 0.001.

CAFs were isolated from the obtained NSCLC tissues to investigate the regulatory role of CAFs in NSCLC. As illustrated in Figure 2A, CAFs marker proteins α‐SMA and FSP1 were confirmed to be positively expressed. Subsequently, we found that lactate production in CAFs was markedly elevated compared to that in fibroblasts sourced from paracancerous lung tissue (Figure 2B). The findings in Figure 2C,D indicated that CAFs induced increased ZNF384 and METTL3 levels. Concurrently, CAFs increased the m6A modification levels of ZNF384 (Figure 2E). Then, we suppressed METTL3 expression in NSCLC cells, and Figure 2F displayed the efficiency of the METTL3 knockdown. As shown in Figure 2G–I, METTL3 deficiency mitigated the stimulatory influence of CAFs on the m6A modification of ZNF384, as well as ZNF384 expression in NSCLC cells. Furthermore, CAFs elevated Pan Kla and H3K18la levels in NSCLC cells (Figure 3A). In addition, CAFs markedly raised the amounts of H3K18la on the METTL3 promoter (Figure 3B). To analyze the lactate secretion by CAFs, cells were treated with Oxamate (lactate generation inhibitor). The results showed that the lactate levels from CAFs supernatant were higher than NF supernatant, and Oxamate treatment remarkedly decreased lactate levels (Figure 3C). More importantly, we observed that the promoting effect of CAFs on METTL3 promoter H3K18la levels and protein expression can be reversed by Oxamate treatment (Figure 3D,E). According to the findings above, lactate released by CAFs stimulated METTL3 expression by H3K18 lactylation of METTL3, and METTL3 upregulated ZNF384 via mediating its m6A modification.

### 3.3 CAFs upregulated ZNF384 expression to induce EMT in NSCLC cells

**FIGURE 4:**
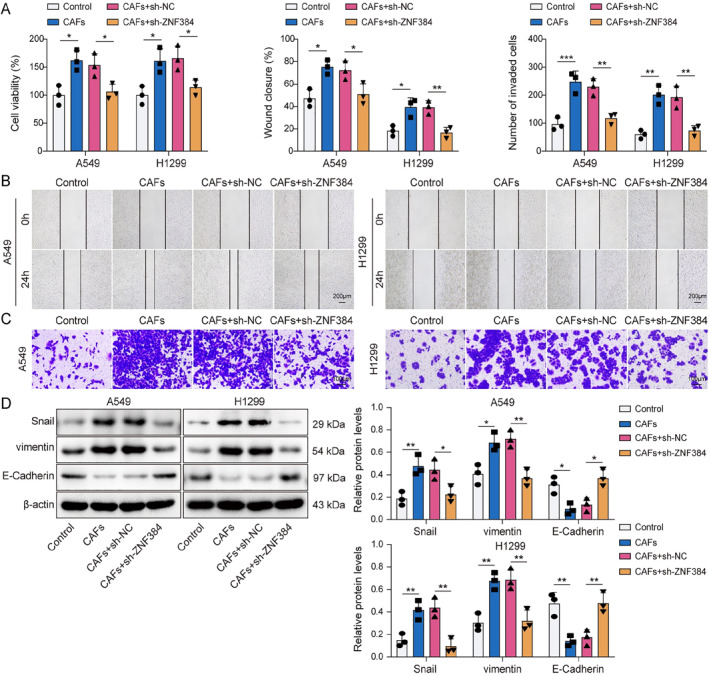
Cancer‐associated fibroblasts (CAFs) upregulated ZNF384 expression to induce epithelial–mesenchymal transition in non‐small cell lung cancer cells. A549 and H1299 cells were treated with conditioned medium from CAFs and were transfected with sh‐NC or sh‐ZNF384 for 24 h. (A) A549 and H1299 cell viability were detected by CCK‐8 assay. (B and C) A549 and H1299 cell migration and invasion were assessed by scratch test and transwell assay. (D) The levels of E‐Cadherin, vimentin, and Snail were determined by Western blot. *p < 0.05, **p < 0.01, ***p < 0.001.

In subsequent investigations, we explored whether CAFs affect the EMT of NSCLC cells through ZNF384. We observed that A549 and H1299 cell viability was enhanced by CAFs, whereas ZNF384 knockdown prevented this effect (Figure 4A). Similarly, ZNF384 knockdown reduced the increase in NSCLC cell migration and invasion caused by CAFs (Figure 4B,C). Furthermore, ZNF384 depletion resulted in a downregulation of vimentin and Snail in NSCLC cells co‐cultured with CAFs, while concurrently elevating E‐Cadherin (Figure 4D). These findings implied that CAFs facilitated the EMT process of NSCLC cells through the modulation of ZNF384.

### 3.4 ZNF384 transcriptionally activated POLR3G

**FIGURE 5:**
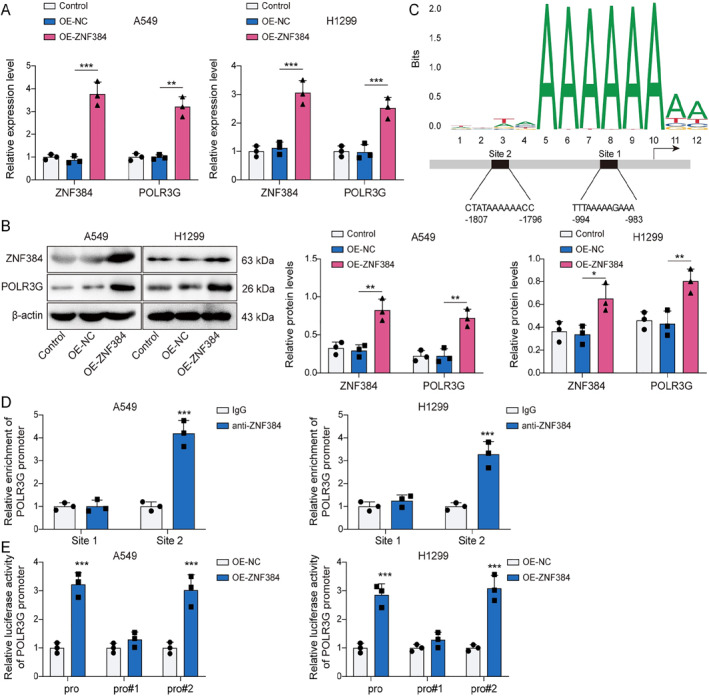
ZNF384 transcriptionally activated POLR3G. A549 and H1299 cells were transfected with OE‐NC or OE‐ZNF384 for 24 h. (A and B) RT‐qPCR and Western blot were conducted to detect ZNF384 and POLR3G expression. (C) JASPAR database (https://jaspar.elixir.no/) predicted the potential binding sites between ZNF384 and POLR3G promoter. (D) ChIP was applied to verify the binding of ZNF384 and POLR3G. (E) Dual‐luciferase reporter assay determined the influence of ZNF384 overexpression on POLR3G transcriptional activity. *p < 0.05, **p < 0.01, ***p < 0.001.

We further investigated the downstream molecules regulated by ZNF284 in NSCLC. Initially, we found that the transfection of OE‐ZNF384 not only caused an increase in ZNF384 expression but also elevated both mRNA and protein levels of POLR3G (Figure 5A,B). As illustrated in Figure 5C, the JASPAR database (https://jaspar.elixir.no/) predicted two potential binding sites between ZNF384 and POLR3G promoter. ChIP assay revealed a remarkable enrichment of ZNF384 at the site2 sequence, confirming that ZNF384 interacted with the POLR3G promoter specifically at this binding site (Figure 5D). Additionally, the overexpression of ZNF384 markedly enhanced the luciferase activity in cells driven by Pro or Pro#2, whereas no such effect was observed with Pro#1 (Figure 5E). The results mentioned above demonstrated that ZNF384 is bound to the POLR3G promoter in NSCLC cells, thereby upregulating its expression.

### 3.5 Knockdown of POLR3G counteracted the promotion effect of ZNF384 on EMT

**FIGURE 6:**
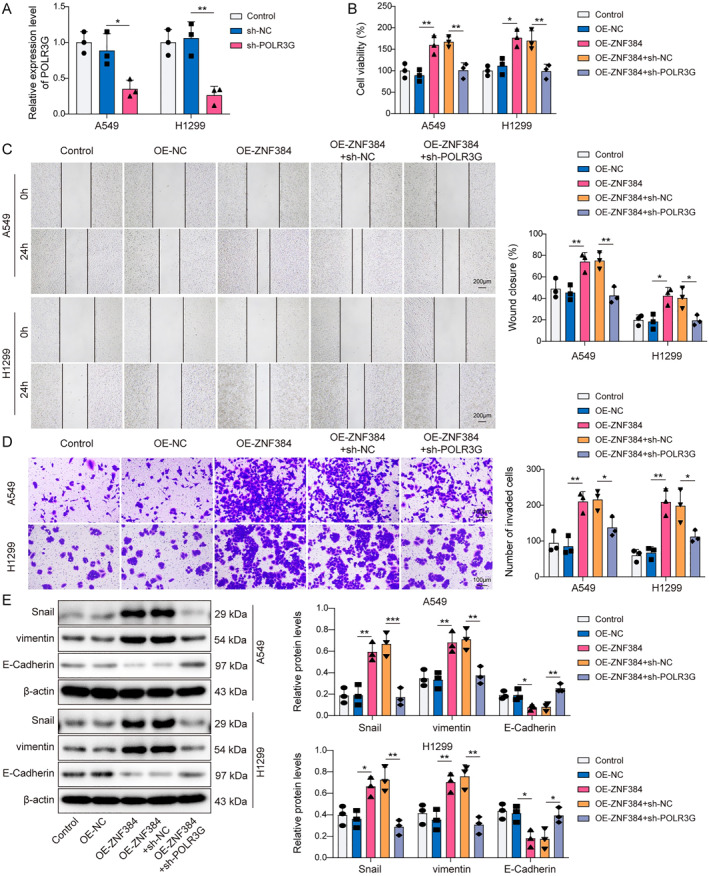
Knockdown of POLR3G counteracted the promotion effect of ZNF384 on epithelial–mesenchymal transition. sh‐NC and sh‐POLR3G were transfected into A549 and H1299 cells for 24 h. (A) RT‐qPCR was conducted to detect POLR3G expression. A549 and H1299 cells were transfected with OE‐NC or OE‐ZNF384 and/or sh‐NC and sh‐POLR3G. (B) CCK‐8 assay was conducted to detect cell viability. (C and D) Scratch test and transwell assay were used to measure cell migration and invasion. (E) Western blot was applied to determine E‐Cadherin, vimentin, and Snail protein levels. *p < 0.05, **p < 0.01, ***p < 0.001.

At last, the function of the ZNF384/POLR3G axis in regulating EMT of NSCLC cells was clarified. As seen in Figure 6A, the transfection of sh‐POLR3G markedly decreased POLR3G levels in A549 and H1299 cells. Further cell functional experiments showed that ZNF384 overexpression led to an increased the viability, migration, and invasion of NSCLC cells (Figure 6B–D). However, POLR3G knockdown abolished the effects induced by ZNF384 (Figure 6B–D). Moreover, POLR3G silencing inhibited the decrease of E‐Cadherin expression and the increase of vimentin and Snail expression caused by ZNF384 overexpression (Figure 6E). In summary, ZNF384 facilitated EMT in NSCLC cells by regulating POLR3G.

## 4 DISCUSSION

Despite the advancements in medical technology that have improved the detection and treatment of NSCLC, patient prognosis remains suboptimal due to issues related to tumor metastasis.^28^ Our study revealed a novel mechanism by which CAFs secreted lactate promoted the transcription of PLORG3 by increasing m6A modification of ZNF384, thereby facilitating the EMT process in NSCLC.

EMT is a biological mechanism that endows cancer cells with the capacity to invade and metastasize, thereby exacerbating tumor malignancy.^29^ ZNF384 has been observed to be elevated in several types of cancers, including colorectal cancer^22^ and breast cancer,^21^ indicating it has carcinogenic potential. Of note, Yang et al. demonstrated that ZNF384 promoted glioma stemness and EMT by increasing the transcriptional of IFI30.^30^ Another study on serous ovarian cancer (SOC) discovered that ZNF384 stimulated the growth of SOC in mice and enhanced the metastatic properties of tumor cells.^31^ Furthermore, research studies have shown that ZNF384 gene mutation in breast cancer tissues and cells may change its gene expression level to delay the course of disease and extend patient survival.^32^ Nevertheless, further research is needed to determine whether the ZNF384 mutation contributes to the occurrence of lung tumors. Our investigation revealed high levels of ZNF384 in NSCLC tissues. Moreover, the inhibition of ZNF384 expression led to an evident reduction in the migration and invasion of NSCLC cells, as well as a suppression of the EMT process. These findings indicated that ZNF384 could be a potential predictive biomarker for NSCLC by controlling the EMT process.

m6A is the most common and conserved co‐transcriptional modification found in eukaryotic RNA, with METTL3 acting as a writer responsible for adding m6A into mRNA.^33^ Our research study found that METTL3 mediated the m6A modification of ZNF384 in NSCLC, which in turn enhanced the expression of ZNF384 and influenced its regulatory role in tumor metastasis. Furthermore, the regulatory influence of METTL3 on ZNF384 was significantly increased following CAFs. CAFs serve as the primary source of lactate within the TME. It had been reported that CAFs influenced the remodeling of the TME in gastric cancer by regulating lactate production and EMT process.^34^ Elevated lactate levels within the TME can drive protein lactylation.^35^ For instance, lactate interacted with AARS1 to lactylate p53 protein, which led to dysregulated activity of p53 and contributed to tumor progression.^36^ Our work reported that lactate secreted by CAFs impacted the H3K18 lactylation of METTL3, thereby enhancing METTL3 expression. Increasing evidence showed that CAFs influence tumorigenesis by altering the biological characteristics of tumor cells.^6^ The considerable heterogeneity observed among CAFs complicates the comprehensive understanding of their specific functions. Research conducted by Hu et al. identified three distinct functional subtypes of CAFs in NSCLC, which were found to impact tumorigenesis by controlling hepatocyte growth factor and FGF7.^37^ We found that CAFs facilitated EMT in NSCLC by regulating ZNF384 expression. Although we did not identify a specific phenotype of CAFs, our results highlight the crucial role of CAFs in the development of NSCLC.

Numerous research studies have confirmed that ZNF384 functioned as a transcription factor that played a role in disease progression by activating its downstream genes. For example, ZNF384 accelerated SOC development by promoting the transcriptional activity of LIN28B.^31^ Notably, we confirmed that ZNF384 is an activating transcription factor for POLR3G in NSCLC. Pol III is the largest RNA polymerase, with POLR3G representing a distinctive subunit.^38^ Research studies have proved a strong association between POLR3G and the metastasis of multiple cancers. Moreover, the knockout of POLR3G markedly diminished the invasive capabilities of triple‐negative breast cancer cells.^39^ Additionally, Liu et al. reported that in transitional cell carcinoma tissues exhibiting elevated POLR3G, there was a significant enrichment of gene sets related to tumor metastasis, including TGF‐β and IL6‐JAK‐STAT3 signaling pathways.^40^ Furthermore, evidence has shown that POLR3G promoted the EMT process in bladder cancer.^26^ Consistent with these findings, our study revealed that the inhibition of POLR3G could counteract the enhancing effect of ZNF384 on the EMT of NSCLC cells, which indicated that the signaling pathway involving ZNF384 and POLR3G facilitated tumor cell migration, invasion, and EMT in NSCLC.

## 5 CONCLUSION

In conclusion, we identified ZNF384 and POLR3G as a potent promoting molecule in NSCLC, they could serve as prognostic biomarkers and treatment targets for NSCLC. Additionally, our research study has elucidated novel molecular mechanisms by which CAFs regulated the EMT process in NSCLC. Specifically, lactate released from CAFs facilitated the H3K18 lactylation of METTL3, which led to ZNF384 upregulation through METTL3‐mediated m6A modification, and ZNF384 subsequently activated POLR3G transcription, ultimately increasing the EMT process of NSCLC cells. Understanding this mechanism shed light on how NSCLC metastasizes and offered new insights and experimental support for the clinical management of advanced NSCLC. However, there are some limitations to this study. This study mainly analyzed the role of ZNF384 at the clinical and cellular levels, and the verification at the animal level will be conducted in the future. Furthermore, the influence of the ZNF384 mutation on the NCSLS process will also be a research direction for us in the future.

## AUTHOR CONTRIBUTIONS

**Ping Li**: Conceptualization; writing—original draft; supervision; methodology; funding acquisition. **Xing Yang**: Validation; investigation. **Hao Tang**: Visualization; resources. **Zhiping Zhou**: Formal analysis; data curation. **Bin Liu**: Writing—review and editing; project administration.

## CONFLICT OF INTEREST STATEMENT

The authors declare no conflicts of interest.

## ETHICS STATEMENT

The protocol approved by the Hunan Aerospace Hospital (No. HNHTYY20240918LLSH‐015‐01). All participants were informed of the purpose and risks of this study and provided informed consent.

## CONSENT FOR PUBLICATION

The informed consent was obtained from study participants.

## Data Availability

The raw data supporting the conclusions of this manuscript will be made available by the authors, without undue reservation, to any qualified researcher.
